# How Much Do Metamemory Beliefs Contribute to the Font-Size Effect in Judgments of Learning?

**DOI:** 10.1371/journal.pone.0142351

**Published:** 2015-11-10

**Authors:** Xiao Hu, Tongtong Li, Jun Zheng, Ningxin Su, Zhaomin Liu, Liang Luo

**Affiliations:** 1 State Key Laboratory of Cognitive Neuroscience and Learning, Beijing Normal University, Beijing, China; 2 School of Sociology, China University of Political Science and Law, Beijing, China; 3 Collaborative Innovation Center of Assessment toward Basic Education Quality, Beijing Normal University, Beijing, China; University College London, UNITED KINGDOM

## Abstract

Evidence shows that the font size of study items significantly influences judgments of learning (JOLs) and that people’s JOLs are generally higher for larger words than for smaller words. Previous studies have suggested that font size influences JOLs in a belief-based way. However, few studies have directly examined how much people’s beliefs contribute to the font-size effect in JOLs. This study investigated the degree to which font size influenced JOLs in a belief-based way. In Experiment 1, one group of participants (learners) studied words with different font sizes and made JOLs, whereas another group of participants (observers) viewed the learners' study phase and made JOLs for the learners. In Experiment 2, participants made both JOLs and belief-based recall predictions for large and small words. Our results suggest that metamemory beliefs play an important role in the font-size effect in JOLs.

## Introduction

Memory monitoring has been a central topic in metamemory research for decades[1–4]. One important form of memory monitoring is the judgment of learning (JOL), which refers to predictions of the likelihood of remembering studied materials in a subsequent memory test [5]. JOLs are important because people rely on their JOLs to guide their learning, such as allocating subsequent study time, choosing items for future study, and terminating the study of mastered items [6–8].Previous studies indicate that JOLs are inferential in nature and are based on a variety of cues [3].One important cue that influences JOLs is the font size of study items. This font-size effect was first reported by Rhodes and Castel [9]. In their research, participants studied words that were presented either in large or small font sizes. The results showed that large words had higher JOLs compared with small words, indicating that participants thought that large words were easier to remember. However, font size did not affect recall performance, which indicated the appearance of a metamemory illusion. This effect of font size on JOLs has been found in many studies [10–13]. The font-size effect indicates that simple perceptual characteristics can influence higher-order cognition and affect judgments about one’s own learning [9].

Although researchers have formed a consensus that font size significantly affects JOLs, the nature of this influence is not clear. Previous studies suggest that the font-size effect in JOLs may be based on people’s metamemory beliefs[12,14].People have beliefs or theories about how a certain cue affects memory performance, and they use these beliefs when making JOLs[15].Previous studies show that beliefs may explain the influence of some cues, such as semantic relatedness and identical pairs, on JOLs[14,16].Mueller et al.[12] recently investigated whether the font-size effect in JOLs is based on beliefs. They asked participants to make belief-based predictions in a questionnaire that described a memory experiment, and they found that people have a belief that large words are easier to remember than small words. This result is consistent with the study by Kornell et al. [11], which also asked participants to make belief-based predictions about large and small words in a questionnaire and showed that people have a belief that large words are easier to remember. In addition, in Experiment 4 of their study, Mueller et al. [12]asked participants to make pre-study JOLs for words with different font sizes and compared the magnitude of the JOLs and the pre-study JOLs. The pre-study JOLs were made before studying each item. Mueller et al. believe that participants cannot learn an item when making pre-study JOLs and that pre-study JOLs should be only based on metamemory beliefs about font size. They found that large words had higher pre-study JOLs than small words, and there was no significant difference between the magnitude of JOLs and pre-study JOLs. According to these results, Mueller et al. [12] suggested that the font-size effect may be based on metamemory beliefs.

Although Mueller et al. [12] suggest that beliefs play an important role in the font-size effect in JOLs, they did not directly examine how much beliefs can explain the font-size effect; rather, they only examined the magnitude of JOLs and belief judgments (predictions in a questionnaire in their Experiment 3a and 3b and pre-study JOLs in their Experiment 4) about different font size. However, previous studies indicate that when investigating whether JOLs are based on beliefs, we should not only examine the influence of a cue on JOLs and belief judgments, but also directly examine the extent to which beliefs explain the variability in JOLs[17,18]. For example, Kornell and Bjork [17] found that when queried directly, participants showed a metamemory belief that more study opportunities lead to better memory performance. However, JOLs were insensitive to the number of study opportunities. Based on these results, Kornell and Bjork [17] suggested that participants failed to use their beliefs about study opportunities when making JOLs. However, Ariel, Hines and Hertzog [18]showed that although there was a significant difference between the influence of study opportunities on JOLs and belief judgments, a regression on the participant level indicated that participants’ beliefs could significantly predict the effect of study opportunities on JOLs, showing that participants did use their beliefs about study opportunities when making JOLs. Similarly, to investigate whether font-size effect in JOLs are based on beliefs, we need to directly examine the extent to which beliefs explain the variability of JOLs.

In this study, we used two different paradigms in two experiments to directly investigate whether beliefs play an important role in the font-size effect in JOLs and the extent to which beliefs explained the font-size effect. In Experiment 1, we used the learner-observer paradigm to investigate whether beliefs play an important role in the font-size effect. One group of participants (observers) viewed the study phase of learners and made JOLs for the learners based on a given cue. The observers could seethe given cue in the learners' study phase but had no access to the learners' processing experience (such as fluency) in relation to the cue. Thus, the influence of the cue on observers’ JOLs should be based on their beliefs. If the given cue significantly affected observers’ JOLs, then we could infer that beliefs may play an important role in the effect of the cue on JOLs. In addition, Experiment 1 asked another group of participants (learners) to study words and make JOLs for themselves based on the given cue. By comparing the influence of the cue on the JOLs of the two groups, we could investigate the influence of beliefs on JOLs, and whether factors other than beliefs (such as fluency) also contributed to the effect of the given cue on JOLs. Previous studies have used the learner-observer paradigm to investigate whether the effect of certain cues (such as retrieval fluency and memory for past test) on JOLs is based on beliefs [19,20]. For example, Matvey et al. [19] used learner-observer paradigm to investigate the influence of retrieval fluency on JOLs. They found that the correlation between retrieval fluency and JOLs did not differ between the learner group and the observer group, which suggested that learners used retrieval fluency in a belief-based way.

In Experiment 2, we asked participants to make belief-based recall predictions in a questionnaire. The questionnaire described an experiment and required the participants to estimate their recall performance for different conditions. By examining the influence of a given cue on JOLs and belief-based predictions, we could investigate whether the cue affected JOLs in belief-based way. Previous studies have often asked separate groups of participants to make JOLs or belief-based predictions [11,12,15].However, this study asked one group of participants to make both belief-based predictions and JOLs. The within-participant design allowed us to conduct a regression analysis on the participant level to directly investigate the degree to which participants' beliefs predicted their JOLs.

To summarize, the present study investigated how much beliefs can explain the font-size effect in JOLs. Experiment 1 used the learner-observer paradigm to examine whether beliefs played an important role in the font-size effect in JOLs. Experiment 2 compared the influence of font size on JOLs and belief-based recall predictions and directly investigated the degree to which font size influenced JOLs in a belief-based way.

## Experiment 1

In Experiment 1, participants were randomly divided into learner and observer groups. Learners studied words with different font sizes and made JOLs for themselves, and observers viewed the learners' study phase and made JOLs for the learners. Observers were aware of the font size for each word in the learners' study phase but were deprived of the experience of processing words with different font sizes. Thus, observers could only use the font size to make JOLs in a belief-based way.

We focused on two results in Experiment 1: the influence of font size on the mean JOL magnitude and the correlation between font size and JOLs in the learner and observer groups. Learners could experience processing words with different font sizes; however, observers had no access to the learners’ processing experience (such as fluency) regarding different font sizes, and thus the influence of font size on observers' JOLs should be based on beliefs. Thus, a significant influence of font size on mean JOL magnitude and a non-zero font-JOL correlation in the observer group would suggest that beliefs may play an important role in the font-size effect in JOLs. In addition, by comparing the font-JOL correlation and the font-size effect on JOL magnitude in the two groups, we could investigate the influence of beliefs on the font-size effect in JOLs.

### Participants

The participants included 40 students from Beijing Normal University (11 men, 29 women). Participants were randomly assigned to either the learner group (*n* = 20) or the observer group (*n* = 20). Each observer was yoked to a learner. Each participant was tested individually, and each participant received 20 RMB (Renminbi, the currency unit of China) as a reward after the experiment. Written informed consent was obtained from all participants. All procedures in this experiment were approved by the Institutional Review Board of the State Key Laboratory of Cognitive Neuroscience and Learning, Beijing Normal University.

### Materials

The materials consisted of 40 Chinese words. All of the words were two-character words that were from the Chinese word database by Cai and Brysbaert [21]. The word frequency was between 3.93 and 46.2 per million words. The 40 words were randomly divided into two sets (each contained 20 words). As in our previous study [10], one set of words was presented in 9-pt font and the other set was presented in 70-pt font. Font size was counterbalanced between participants. The two sets of words did not differ in the word frequency or the number of strokes (*p*> .3).

### Procedure

The experimental procedures for learners and observers differed.

#### Learners

The learners took part in a free recall task that consisted of three phases: the study phase (in which JOLs were made), the distractor task phase, and the memory test phase. In the study phase, learners were required to study 40 words with a 4-s presentation time for each word. Immediately following the presentation of each word, learners were instructed to type a number from 0 to 100 that represented the probability of recalling that word in a memory test, which took place one minute after studying all the words. Words were presented in a fixed random order in which no more than three words of the same font size were presented consecutively. After the study phase, learners engaged in a one-minute arithmetic distractor task. They were then given a recall test in which they were instructed to type as many of the items as they could remember from the study list. There was no time limit for the recall test.

#### Observers

Observers first read the following description of the free recall task for learners:

We conducted an experiment in which students were asked to remember a list of two-character Chinese words. They were presented with 40 words, one after another. Each of the words was presented for 4 s. Half of the words (20) were presented in a large font size, and half of the words (20) were presented in a small font size. Students were asked to try to remember as many words as possible. One minute after studying all the words, a memory test took place in which students were required to recall as many previously studied words as they could. There was no time limit for the memory test.

The computer screen then presented two rectangles to illustrate the size of the two-character Chinese words in 70-pt font and 9-pt font. The rectangles removed the possibility of experiencing the processing of words in different font sizes [12].After seeing the rectangles, observers were informed that another participant (a learner) had already completed the task and that they were required to make predictions about the learner's probability of recalling each word in the memory test. Importantly, observers were told that they were not to study the items themselves but to simply make predictions about the learner’s performance. Observers saw each word for 4 s in the same order as their yoked learner did. The words were presented on the computer screen in the same font size of 33-pt, and the word "large" or "small" was presented along with each word to indicate the font size that the yoked learner saw when he or she studied that word. Immediately following the presentation of each word, observers were instructed to type a number from 0 to 100 that represented the learner's probability of recalling that word in the memory test.

### Results and discussion

**Fig 1:**
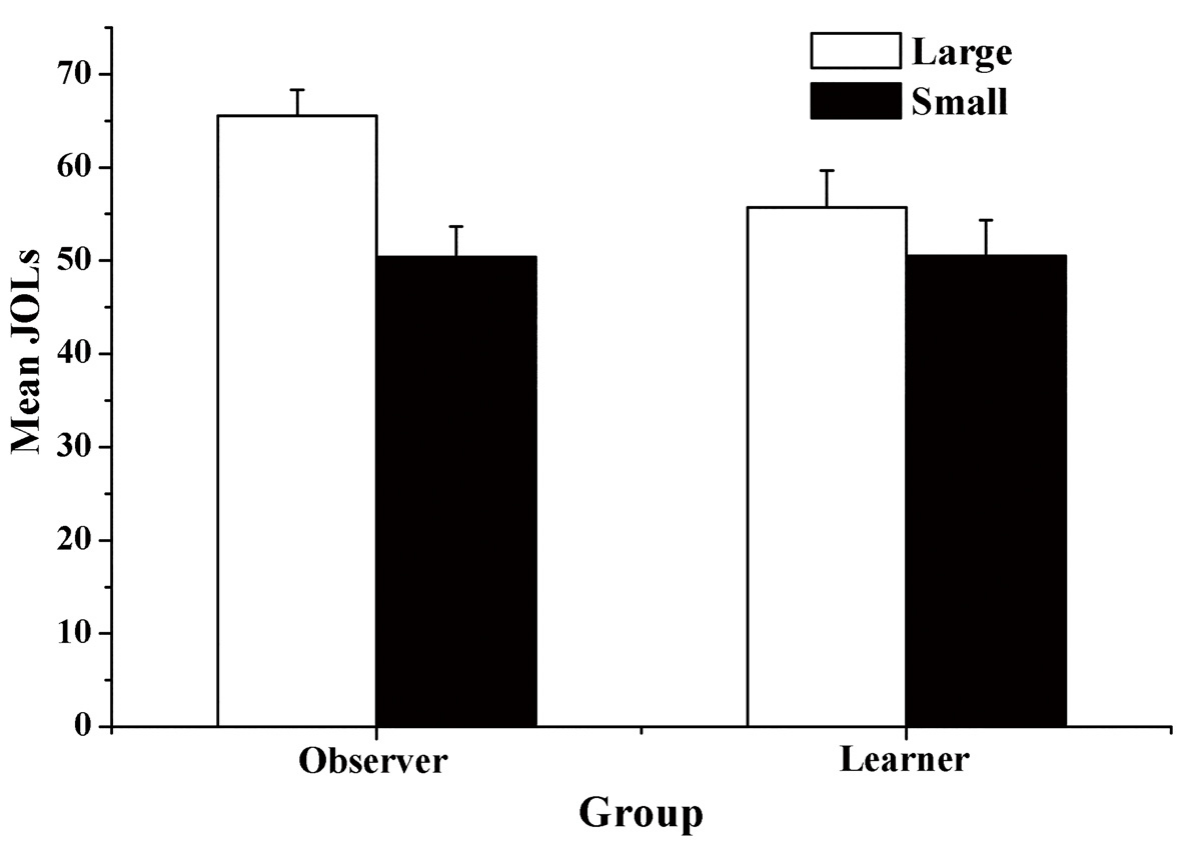
Mean JOL magnitude as a function of font size and group in Experiment 1. Error bars represent standard errors.

The mean JOL magnitude in the learner group was significantly higher for large words than for small words, *t* (19) = 2.58, *p* = .018, *d* = 0.58. Similarly, the mean JOL magnitude in the observer group was significantly higher for large words than for small words, *t* (19) = 5.85, *p*< .001, *d* = 1.31. More importantly, the difference in JOLs between large and small words was significantly larger in the observer group than in the learner group, *t* (38) = 3.04, *p* = .004, *d* = 0.96 (see Fig 1).

**Fig 2:**
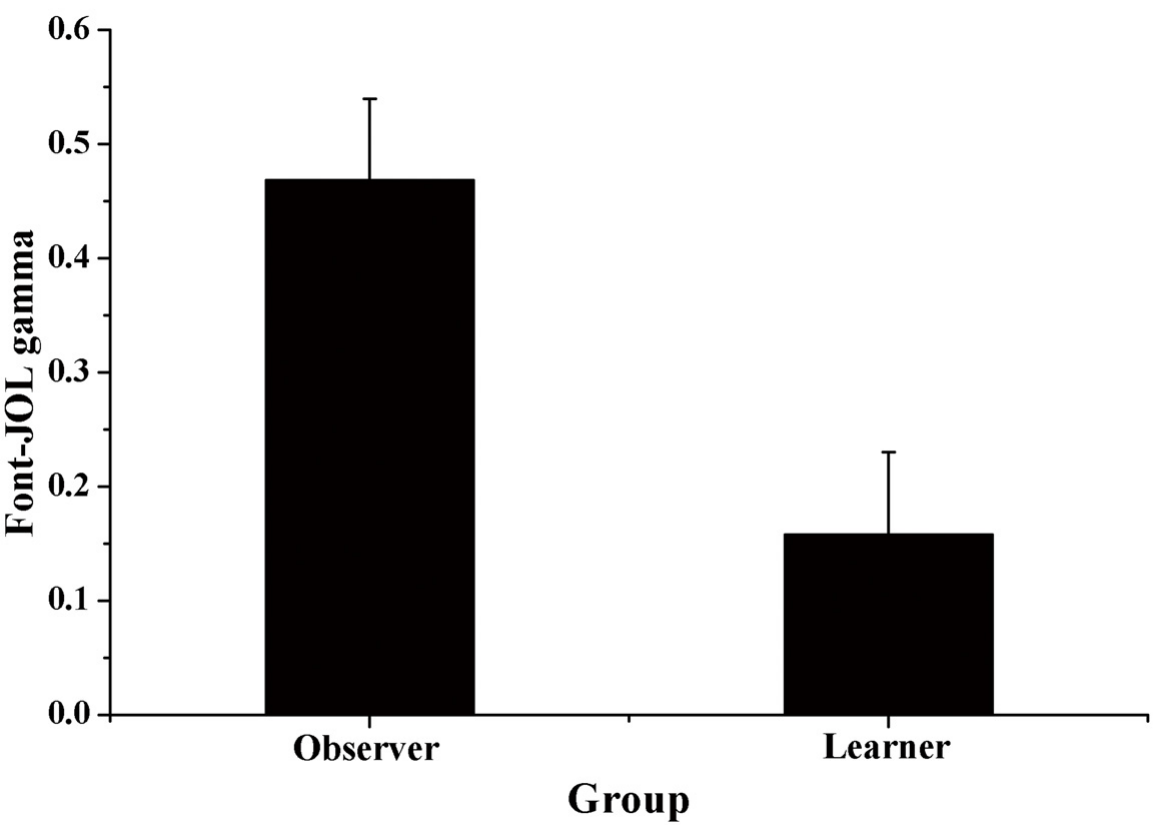
Font-JOL gamma correlation as a function of group in Experiment 1. Error bars represent standard errors.

We then calculated the Goodman-Kruskal gamma correlation [22] between the font size and JOLs for each participant and compared the gamma correlation between the two groups [19]. The font-JOL correlation for both the learner and the observer groups was significantly higher than zero, *t*_*learner*_ (19) = 2.23, *p* = .038, *d* = 0.50; *t*_*observer*_ (19) = 6.49, *p*< .001, *d* = 1.45. In addition, the font-JOL correlation was significantly higher in the observer group than in the learner group, t (38) = 3.07, *p* = .004, *d* = 0.97 (see Fig 2).

We also compared the recall performance and relative accuracy (as measured by the Goodman-Kruskal gamma correlation between the JOL magnitude and recall performance; see [22]) of large and small words in the learner group. Recall performance did not differ between large and small words, *t* (19) = 0.74, *p* = .469, *d* = 0.17. The relative accuracy for both large and small words was significantly higher than zero, *t*_*large*_ (19) = 3.70, *p* = .002, *d* = 0.83; *t*_*small*_ (19) = 2.99, *p* = .008, *d* = 0.67. In addition, the relative accuracy did not differ between different font sizes, *t* (19) = 0.23, *p* = .821, *d* = 0.05 (see Table 1; see S1 Data for the data collected in Experiment 1).

**Table 1:** Recall performance and relative accuracy from Experiments 1–2.

	Recall Performance		Relative Accuracy	
	Large	Small	Large	Small
Experiment 1	54.00 (15.61)	51.75 (14.08)	0.22 (0.27)	0.21 (0.31)
Experiment 2	47.00 (18.03)	49.60 (18.76)	0.28 (0.32)	0.28 (0.33)

**Note.** Standard deviations are reported in parentheses. Recall performance refers to the percentage of recalled words. Recall performance and relative accuracy in Experiment 1 are from learner group.

Experiment 1 showed that mean JOL magnitude for large words was significantly higher than that for small words in the observer group and that the font-JOL correlation for the observer group was significantly higher than zero. These results indicated that beliefs played an important role in the font-size effect in JOLs. In addition, we found that the influence of font size on JOLs was significantly larger in the observer group than in the learner group. However, while the results of Experiment 1 indicated that metamemory beliefs may play an important role in the font-size effect in JOLs, they could not show the extent to which beliefs explain the font-size effect. Thus, in Experiment 2, we directly examined the degree to which beliefs explained the font-size effect in JOLs.

## Experiment 2

In Experiment 2, we directly investigated the extent to which beliefs contributed to the font-size effect in JOLs. Participants in Experiment 2 made both belief-based recall predictions in a questionnaire and JOLs for large and small words. We hypothesized that belief-based recall predictions should significantly differ between different font sizes and that participants should have a belief that large words are easier to remember than small words[12]. In addition, a regression analysis was conducted to examine the extent to which the participants' beliefs contributed to the font-size effect in JOLs. If beliefs play an important role in font-size effect in JOLs, then beliefs about font size should significantly predict the variability of the font-size effect in JOLs.

### Participants

The participants included 25 students from Beijing Normal University (6 men, 19 women). Each participant was tested individually, and each participant received 25 RMB as a reward after the experiment. Written informed consent was obtained from all participants. All procedures in this experiment were approved by the Institutional Review Board of the State Key Laboratory of Cognitive Neuroscience and Learning, Beijing Normal University.

### Materials

The materials were the same as those used in Experiment 1.

### Procedure

The experiment took place over two days and consisted of two separate tasks, the belief judgment task and the free recall task. There was one day between the two tasks. On the first day, participants took part in the belief judgment task. They read the description of a free recall task and saw the rectangles that represented different font sizes, as the observers did in Experiment 1. They were then asked to estimate the number of words (out of 20) of each font size that the students in the free recall task would recall. The order of estimates for each font size was counterbalanced across participants.

Participants left the lab after finishing the belief judgment task. They returned 24 hours later to take part in the free recall task, which was the same as the task for learners in Experiment 1.

### Results and discussion

**Fig 3:**
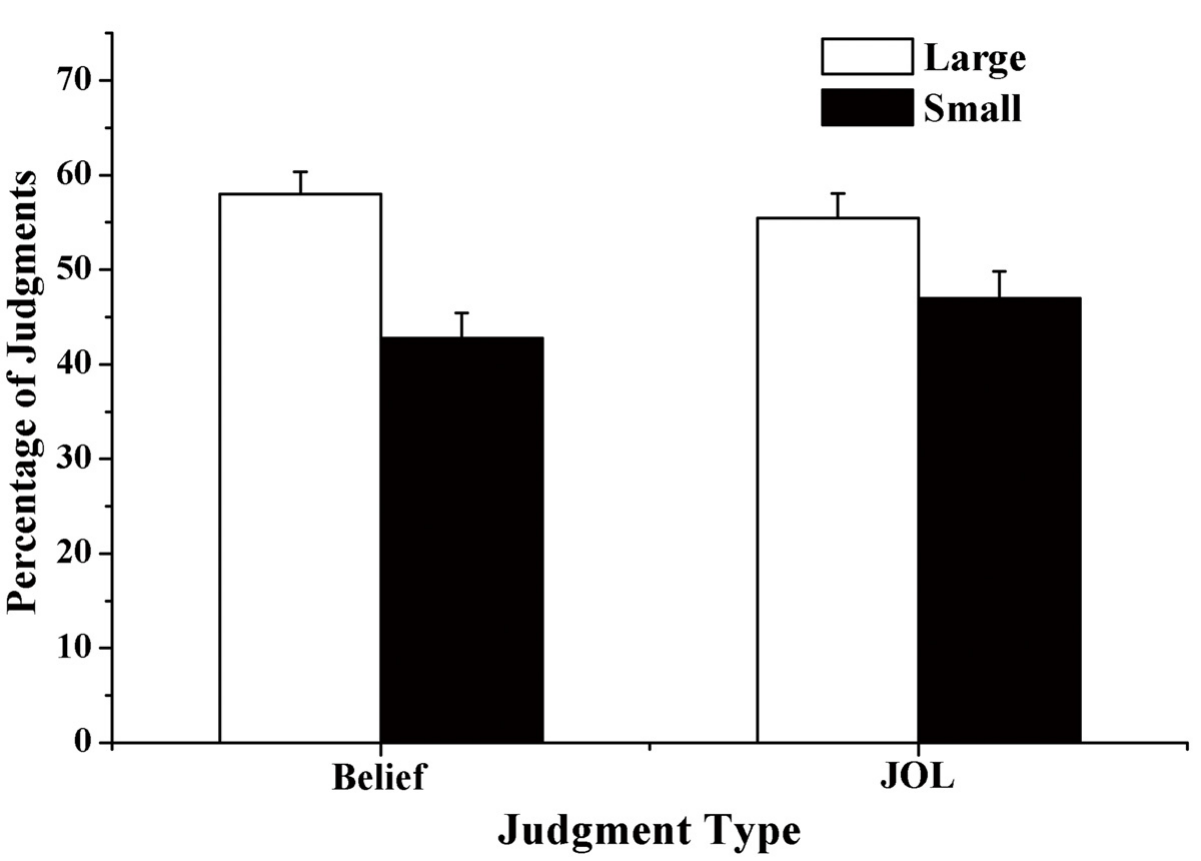
Percentage of judgments as a function of font size and judgment type in Experiment 2. Error bars represent standard errors.

Estimates in the belief judgment task were transformed into percentages and then compared between different font sizes. The participants believed that memory performance would be significantly higher for large words than for small words, *t* (24) = 6.32, *p*< .001, *d* = 1.26. Similarly, the mean JOL magnitude in the free recall task was significantly higher for large words than for small words, *t* (24) = 5.66, *p*< .001, *d* = 1.13. More importantly, the difference between large and small words was significantly larger in belief-based predictions than in JOLs, *t* (24) = 3.15, *p* = .004, *d* = 0.63 (see Fig 3).

**Fig 4:**
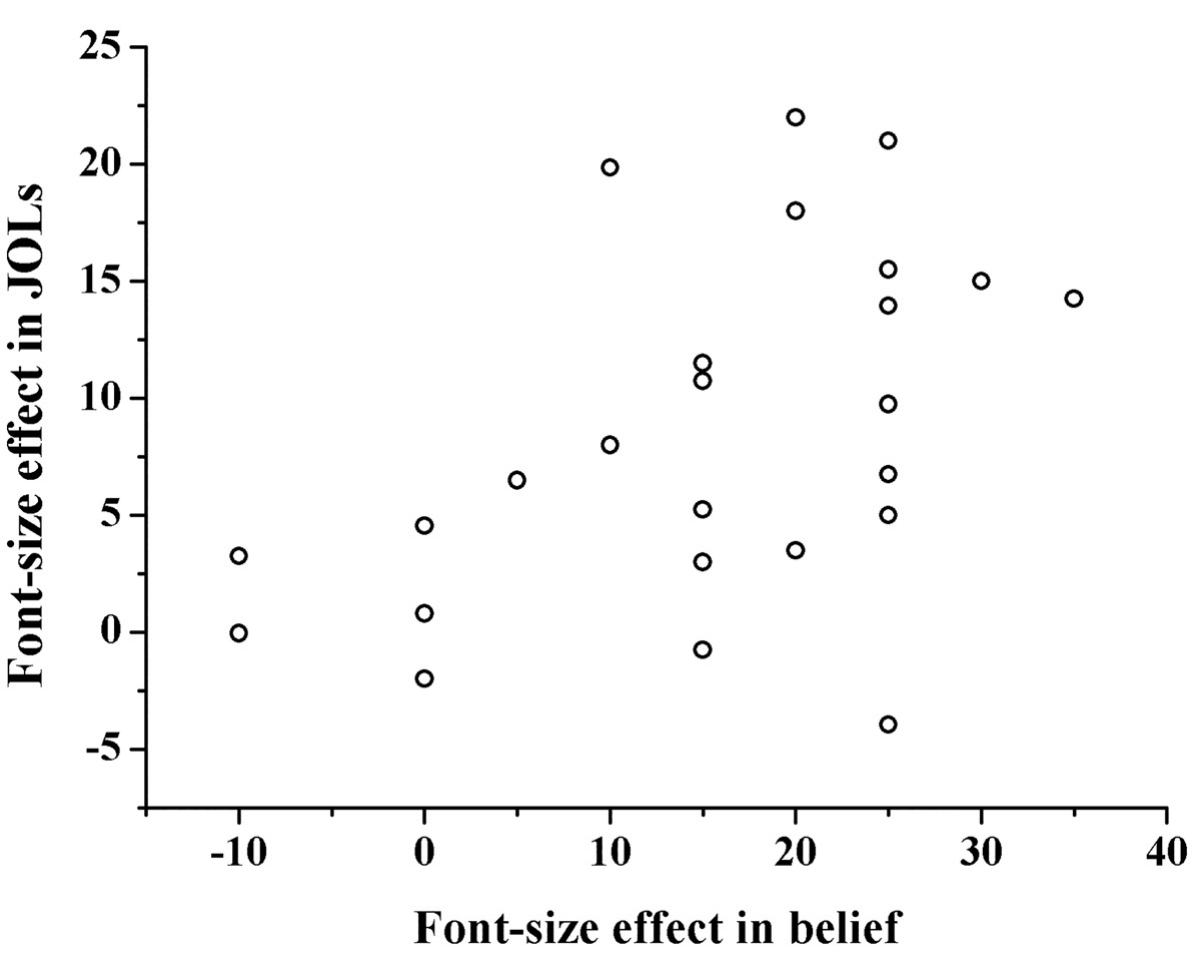
The relationship between font-size effect in belief and in JOLs in Experiment 2. The font-size effect is the difference in percentage of judgments between large and small words. Each point represents one individual participant.

We then conducted a regression analysis on the participant level in which the difference in the mean JOL magnitude between large and small words was regressed on the difference in belief-based predictions between large and small words (see Fig 4). The results showed that difference in belief-based predictions between large and small words significantly predicted the difference in mean JOL magnitude between large and small words, *F* (1, 23) = 6.81, *p* = .016, *R* ^*2*^ = .228, adjusted *R* ^*2*^ = .195.

We also compared the recall performance and relative accuracy between large and small words in the free recall task (see Table 1). Recall performance did not differ between large and small words, *t* (24) = -1.18, *p* = .249, *d* = 0.24. Relative accuracy for both large and small words was significantly higher than zero, *t*_*large*_ (23) = 4.39, *p*< .001, *d* = 0.90; *t*_*small*_ (23) = 4.18, *p*< .001, *d* = 0.85. In addition, the relative accuracy did not differ between different font sizes, *t* (23) <0.01, *p* = .998, *d*<0.01 (see S2 Data for the data collected in Experiment 2.One female participant in Experiment 2 recalled all forty words in the memory test. Her data were excluded from the relative accuracy analysis, which is reflected in the variations in the degrees of freedom that are reported for the statistical tests).

The results from Experiment 2 suggested that participants had a belief that large words are easier to remember than small words, and participants' beliefs significantly explained the variability of the font-size effect in JOLs. In addition, similar to Experiment 1, Experiment 2 found that the font-size effect was significantly larger in the belief-based predictions than in the JOLs.

## General Discussion

The current study investigated the degree to which font size influenced JOLs in a belief-based way. Experiment 1 used the learner-observer paradigm, and the results showed that observers' JOLs were significantly higher for large words than for small words. In addition, the influence of font size on JOLs was significantly larger in the observer group than in the learner group. Experiment 2 asked participants to make belief-based predictions in a questionnaire and JOLs for large and small words. The results showed that belief-based predictions were significantly higher for large words than for small words and, as in Experiment 1, the difference between large and small words was significantly larger for the belief-based predictions than for the JOLs. In addition, the regression analysis indicated that beliefs significantly explained the variability of the font-size effect in JOLs.

Similar to previous studies[11,12], the current study provided evidence that people have a belief that large words are easier to remember than small words. We found that participants had beliefs about font size in both experiments and indifferent paradigms. The results in Experiment 1 showed that font size significantly influenced JOLs in the observer group. Observers had no access to the learners’ processing experiences of different font sizes; therefore, the influence of font size on observers' JOLs should have been based on beliefs. Thus, the significant font-size effect in the JOLs of observer group suggests that people have beliefs about font size and that those beliefs may play an important role in font-size effect in JOLs. In addition, our Experiment 2 indicated that in a belief-based questionnaire, participants’ recall prediction for large words was significantly higher than that for small words. Our experiments support previous studies [11,12] that also showed that people have a belief that large words are easier to remember.

In addition, the regression analysis in Experiment 2 showed that the font-size effect in belief-based predictions can significantly explain the variability of JOLs. In contrast with previous study that only compared the effect of font-size on JOLs and belief-based predictions [12], the regression analysis in our study provided direct evidence that beliefs about font size did contribute to the font-size effect in JOLs. To the best of our knowledge, our study is among the first to directly investigate the degree to which beliefs explain the font-size effect. We suggest that to examine the effect of beliefs on the font-size effect in JOLs, we should not only investigate the font-size effect in JOLs and belief-based predictions, but also conduct regression analyses between beliefs about font size and JOLs.

Moreover, we must note that while our results suggest that beliefs played an important role in font-size effect in JOLs, we could not accurately assess the extent to which beliefs contribute to the font-size effect because some uninteresting causes derived from our experimental paradigms may reduce the degree to which beliefs explain the variability of JOLs. For example, in Experiment 2, the belief-based predictions and JOLs were made on different days, which could have resulted in intra-participant noise and reduced the correlation between belief-based predictions and JOLs. Another example is that Experiment 2 asked participants to make global belief-based predictions and item-by-item JOLs. Participants could not see individual words when making global belief-based predictions and may have based their predictions mainly on abstract beliefs. However, item-by-item JOLs might be based more on the characteristics of individual items. Thus, global belief-based predictions may only partly explain the influence of beliefs on the font-size effect in item-by-item JOLs. Future research should reduce or eliminate these uninteresting causes and accurately assess the degree to which beliefs contribute to the font-size effect in JOLs.

Another interesting finding was that in both experiments, the influence of font size was significantly larger in belief-based predictions than in JOLs. This finding was similar to the findings of Kornell et al. [11] and Mueller et al. [12], both of which indicated that the font-size effect was numerically larger in beliefs than in JOLs, although neither of the studies statistically compared the influence of font size on belief-based predictions and JOLs. In addition, we statistically compared the influence of font size on JOLs and belief-based predictions using the data from Experiment 1 and Experiment 3 of Kornell et al. [11], which revealed that the font-size effect was significantly larger in belief-based predictions than in JOLs (*p* < .05). These results suggest that in addition to beliefs, other factors might also contribute to the font-size effect in JOLs. One possibility is that compared with belief-based predictions, JOLs may be based more on multiple cues other than font size, such as the frequency of individual words; this may lead to a smaller font-size effect in JOLs than in belief-based predictions even when the font-size effect in JOLs was fully based on beliefs. Another factor that may contribute to the font-size effect is fluency. Fluency is the subjective experience of ease with which people process information [23]. Some researchers suggest that processing large words is more fluent than processing small words, which may lead to higher JOLs for large words than for small words [9,13]. Our study did not provide evidence about the influence of fluency on font-size effect in JOLs because the results of Experiment 1 showed that the font-size effect was significantly larger in observer's JOLs than in learners' JOLs. According to Matvey et al. [19], if both beliefs and fluency contribute to the font-size effect in JOLs, then the font-size effect in observers' JOLs should be smaller than the effect in learners' JOLs. However, there is a possibility that the effect of fluency and beliefs on JOLs may not simply add together when people make JOLs for words with different font sizes. Instead, considering the effect of fluency on JOLs may make people rely less on beliefs. Thus, if the influence of fluency on the font-size effect in JOLs is smaller than the influence of beliefs, then the consideration of both fluency and beliefs could lead to a smaller font-size effect in JOLs than in belief-based predictions. In addition, although Mueller et al. [12] suggest that fluency does not differ between large and small words, they measured only two types of fluency: response time in a lexical decision task and study time in self-paced study. There may be other types of fluency that differ significantly between large and small words [24]. For example, although there is no significant difference in the study time for large and small words in self-paced study, previous research shows that people’s subjective estimations of study time can differ from their real study time [25]. Thus, the subjective estimation of study time may differ significantly between words with different font sizes. Future studies should investigate in greater detail whether words with large and small font sizes differ significantly in people’s subjective estimations of the study time or other types of processing fluency. In addition, future studies should measure fluency and beliefs about different font sizes in one experiment and should conduct multiple regression analyses to examine the degree to which beliefs and fluency independently contribute to the font-size effect in JOLs.

## Supporting Information

S1 Data Data collected in Experiment 1. (SAV)

S2 Data Data collected in Experiment 2. (SAV)
